# 2,5-Dihydroxybenzoic Acid Ameliorates Metabolic Dysfunction-Associated Steatotic Liver Disease by Targeting the CCL2-CCR2 Axis to Reduce Lipid Accumulation

**DOI:** 10.3390/nu17111835

**Published:** 2025-05-28

**Authors:** Chien-Yun Hsiang, Kuang-Ting Hsu, Hsin-Yi Lo, Yun-Jhu Hou, Tin-Yun Ho

**Affiliations:** 1 Department of Microbiology and Immunology, China Medical University, Taichung 404333, Taiwan; cyhsiang@mail.cmu.edu.tw; 2 School of Medicine, China Medical University, Taichung 404333, Taiwan; u109001433@cmu.edu.tw; 3 Graduate Institute of Chinese Medicine, China Medical University, Taichung 404333, Taiwan; hsinyilo0123@gmail.com; 4 Department of Animal Science and Technology, National Taiwan University, Taipei 106319, Taiwan; b13606015@ntu.edu.tw; 5 Department of Health and Nutrition Biotechnology, Asia University, Taichung 413305, Taiwan

**Keywords:** metabolic dysfunction-associated steatotic liver disease, 2,5-dihydroxybenzoic acid, chemokine (C-C motif) ligand 2, chemokine (C-C motif) receptor 2

## Abstract

**Background/Objectives:** Metabolic dysfunction-associated steatotic liver disease (MASLD) is the most prevalent chronic liver disease worldwide, contributing to metabolic dysfunction and increased healthcare costs. The green Mediterranean diet reduces intrahepatic fat and elevates the plasma levels of 2,5-dihydroxybenzoic acid (2,5-DHBA), suggesting a mechanistic role for 2,5-DHBA in hepatic lipid metabolism. This study aimed to evaluate the therapeutic potential of 2,5-DHBA in MASLD and elucidate its molecular mechanism. **Methods:** Lipid accumulation was assessed in oleic acid-treated HepG2 cells and a high-fat diet (HFD)-induced MASLD mouse model. RNA sequencing, molecular docking, and immunohistochemical staining were performed to investigate the molecular mechanisms, focusing on the chemokine (C-C motif) ligand 2 (CCL2)–CCL2 receptor (CCR2) axis. **Results:** 2,5-DHBA significantly reduced hepatic lipid accumulation in both HepG2 cells and HFD-fed mice in a dose-dependent manner. RNA sequencing revealed the marked downregulation of CCL2, a key proinflammatory mediator in MASLD pathogenesis. Molecular docking predicted that 2,5-DHBA competed with CCL2 for binding at the CCR2 axis. Immunohistochemistry further confirmed that 2,5-DHBA treatment lowered hepatic CCL2 expression, suppressed nuclear factor-κB activation, and reduced inflammatory cell infiltration. These findings suggest that 2,5-DHBA exerted anti-steatotic effects by modulating the CCL2-CCR2 signaling pathway. **Conclusions:** This is the first study to demonstrate that 2,5-DHBA attenuates hepatic steatosis via targeting the CCL2-CCR2 axis. These findings highlight its potential as a novel nutraceutical strategy for MASLD treatment.

## 1. Introduction

Metabolic dysfunction-associated steatotic liver disease (MASLD) is the most common liver disease worldwide [1,2]. A systematic review and meta-analysis of the global incidence of MASLD reveals that its prevalence increased from 25.26% between 1990 and 2006 to 38% from 2016 to 2019, representing a 50.4% rise over this decade [3]. Nutritional and dietary interventions play a critical role in counteracting the growing burden of MASLD [4,5]. Diets high in fructose, saturated fat, sugar-sweetened beverages, and ultra-processed foods are associated with the development of MASLD. Conversely, low-carbohydrate diets, high-fiber diets, and those rich in monounsaturated and polyunsaturated fatty acids have shown benefits in managing the disease [4,6,7]. Among these strategies, the Mediterranean diet is the most extensively studied intervention for MASLD management [8,9,10].

The Mediterranean diet has been shown to improve insulin resistance, reduce hepatic fat accumulation, and mitigate angiogenesis-related pathologies [4,11,12,13]. Adherence to this diet is inversely associated with the prevalence and severity of MASLD [14,15]. An 18-month randomized clinical trial, the Dietary Intervention Randomized Controlled Trial Polyphenols Unprocessed (DIRECT PLUS), demonstrates that the green Mediterranean diet, which is enriched with fruits and vegetables and reduced in red and processed meat, results in twice the reduction in intrahepatic fat compared to other healthy dietary strategies and halves the MASLD incidence. Participants following the green Mediterranean diet for 18 months exhibited significantly higher plasma levels of total polyphenols, particularly 2,5-dihydroxybenzoic acid (2,5-DHBA), compared to those on other healthy diets [16]. These findings suggest a correlation between reduced intrahepatic fat and elevated blood levels of 2,5-DHBA in individuals adhering to the green Mediterranean diet.

2,5-DHBA is a polyphenol that is widely present in various fruits and vegetables, including cranberries, grapes, citrus fruits, kale, and lotus seeds. It exhibits a broad range of health benefits, such as anti-inflammatory, antioxidant, antimicrobial, cardioprotective, neuroprotective, and nephroprotective effects [17]. Supplementation with 2,5-DHBA has been shown to prevent obesity induced by high-fat and high-fructose diets in mice by enhancing fatty acid oxidation and stimulating thermogenesis in brown adipose tissue [18]. These findings raise the following question: does 2,5-DHBA, a polyphenol metabolite that is elevated in individuals following a green Mediterranean diet, confer specific benefits that improve MASLD? To explore this, we analyzed the effects of 2,5-DHBA on lipid accumulation in an oleic acid (OA)-induced HepG2 cell model and a high-fat diet (HFD)-induced MASLD mouse model. Gene expression changes induced by 2,5-DHBA were assessed via RNA sequencing (RNA-Seq) to elucidate its mechanisms of action. Molecular docking and immunohistochemical (IHC) staining were used to verify the potential targets. Our findings suggest that 2,5-DHBA disrupted the chemokine (C-C motif) ligand 2 (CCL2)–CCL2 receptor (CCR2) axis, leading to reduced hepatic lipid accumulation in both OA-induced HepG2 cells and HFD-fed mice.

## 2. Materials and Methods

### 2.1. Chemicals

All chemicals, unless otherwise indicated, were purchased from Sigma-Aldrich (St. Louis, MO, USA). OA and simvastatin were dissolved in dimethyl sulfoxide (DMSO) at final concentrations of 200 mM and 10 mM, respectively. 2,5-DHBA was dissolved in DMSO at a final concentration of 10 mM. Oil Red O was dissolved in isopropanol at a concentration of 5 g/L. The Cell Counting Kit-8 (CCK-8) was obtained from MedChemExpress (Monmouth Junction, NJ, USA). Rabbit polyclonal antibody against CCL2 (ab25124) and rabbit monoclonal antibody against CD11b (ab133357) were purchased from Abcam (Cambridge, MA, USA). Mouse monoclonal antibody against p65 (MAB3026) was purchased from Millipore (Burlington, MA, USA).

### 2.2. Cell Culture and OA-Induced Cell Model

Human hepatoblastoma cells (HepG2) were purchased from the Bioresource Collection and Research Center (Hsinchun, Taiwan). HepG2 cells were cultured in Dulbecco’s modified Eagle’s medium (DMEM) (Hyclone, Logan, UT, USA) supplemented with 10% fetal bovine serum (Sigma-Aldrich, St Louis, MO, USA) and 1% penicillin–streptomycin (Invitrogen, Carlsbad, CA, USA) in a humidified incubator with 5% CO_2_ at 37 °C.

To induce intracellular lipid accumulation, HepG2 cells were seeded in a 6-well plate and grown to 80% confluence. DMEM containing 0.5 mM of OA was then added to the wells and they were incubated for 24 h. The final concentration of DMSO in each well was 0.1%.

### 2.3. Cell Viability Assay

HepG2 cells were seeded into 96-well plates at a density of 1 × 10^5^ cells/well and incubated at 37 °C for 24 h. Subsequently, the cells were treated with DMEM containing various concentrations of OA, or with DMEM containing 0.5 mM OA and different concentrations of 2,5-DHBA, for an additional 24 h. Following the treatment period, a 1/10 volume of CCK-8 solution was added to each well and they were incubated at 37 °C for 2 h. The optical density (OD) of each well was then measured at 450 nm using a microplate reader (Multiskan GO, Thermo Scientific, Waltham, MA, USA). Cell viability (%) was calculated as (OD of the drug treatment group/OD of the no-drug treatment group) × 100%.

### 2.4. Oil Red O Staining

HepG2 cells cultured in 6-well plates were induced with 0.5 mM OA for 24 h and subsequently treated with 10 μM simvastatin or various concentrations of 2,5-DHBA in the presence of 0.5 mM OA for an additional 24 h. Cells were then washed with phosphate-buffered saline (PBS) (137 mM NaCl, 1.4 mM KH_2_PO_4_, 4.3 mM Na_2_HPO_4_, 2.7 mM KCl, pH 7.2), fixed in 10% formaldehyde solution for 1 h, and washed with 60% isopropanol. Subsequently, cells were stained with Oil Red O working solution (Oil Red O stock solution–distilled water = 3:2) at room temperature for 10 min, washed four times with distilled water, and imaged using a camera. For quantification, 100% isopropanol was added to each well and they were incubated at room temperature for 10 min to dissolve the Oil Red O. The OD of each well was then measured at 520 nm using a microplate reader (Multiskan GO, Thermo Scientific, Waltham, MA, USA).

For liver tissue, fresh samples weighing approximately 30 mg were washed with PBS and stained with Oil Red O working solution at room temperature for 120 min. After staining, the samples were washed with distilled water to remove excess dye, homogenized in 100% isopropanol to dissolve the Oil Red O, and centrifuged at 12,000× *g* for 15 min at 4 °C to remove tissue debris. The supernatant was collected and transferred to a 96-well plate. The OD of each well was measured at 520 nm using a microplate reader (Multiskan GO, Thermo Scientific, Waltham, MA, USA).

### 2.5. MASLD Animal Experiment

Female FVB mice, aged five weeks and weighing 20 ± 1 g, were housed at the China Medical University Animal Center. The mice had *ad libitum* access to food and water and were kept in a controlled environment with a 12 h light/dark cycle. The animal experiment protocol was approved by the Institutional Animal Care and Use Committee of China Medical University (Permit No. CMUIACUC-2022-456) and conducted in accordance with the U.S. Guidelines for the Care and Use of Laboratory Animals (NIH Publication No. 85-23, revised 1996). For the short-term experiment, mice were randomly divided into five groups (five mice per group): a mock group fed with a normal diet (10% energy from fat, D12450B, Research Diet, New Brunswick, NJ, USA), an HFD group that received a high-fat diet (60% energy from fat, 58Y1, TestDiet, St Louis, MO, USA), and 2,5-DHBA groups fed with an HFD and orally administered 2,5-DHBA at 1, 10, or 100 mg/kg once daily by oral gavage for 7 days. The mock and HFD groups received the same volume of distilled water daily for 7 days.

For the long-term experiment, mice were randomly divided into three groups (ten mice per group): a mock group fed with a normal diet, an HFD group that received a high-fat diet, and a 2,5-DHBA group fed with a high-fat diet and treated with 2,5-DHBA (100 mg/kg/day orally). Mice were fed their respective diets for 4 months. 2,5-DHBA administration began in the fourth month and continued for 4 weeks. The mock and HFD groups received distilled water daily during the fourth month.

At the end of the experiment, mice were sacrificed under 4% isoflurane (Baxter, Guayama, PR, USA), and liver tissue was collected for analysis.

### 2.6. RNA-Seq and Pathway Analysis

Total RNA was extracted from liver tissue (30 mg) using the RNeasy Mini Kit (Qiagen, Valencia, CA, USA). The RNA concentration and integrity were assessed using an Agilent 2100 bioanalyzer (Santa Clara, CA, USA). RNA-Seq was performed as previously described [19]. Paired-end sequencing (150 bp) was performed using an Illumina NovaSeq 6000 (Illumina, San Diego, CA, USA). Significant differentially expressed genes (DEGs) were defined as those with fold changes ≥ 1.5 or ≤ −1.5 and an adjusted *p*-value ≤ 0.05. A Kyoto Encyclopedia of Genes and Genomes (KEGG) pathway analysis was performed using NetworkAnalyst (https://www.networkanalyst.ca/) [20]. Volcano plots were generated using Microsoft Excel 2021 based on the log_2_ fold change and –log_10_ *p*-value.

### 2.7. Molecular Docking Analysis of CCR2 and 2,5-DHBA

The molecular docking analysis was performed using PatchDock (https://www.cs.tau.ac.il//~ppdock/PatchDock/ (accessed on 1 October 2024)) [21]. The structure of CCR2 (PDB ID: 1KAD) was obtained from the Protein Data Bank (https://www.rcsb.org/) and used as the target for docking with 2,5-DHBA. The structure of 2,5-DHBA was obtained from ZINC (https://zinc.docking.org/ (accessed on 15 September 2024)) [22]. The docking structure was generated and visualized using UCSF Chimera X (https://www.cgl.ucsf.edu/chimerax/ (accessed on 25 October 2024)) [23].

### 2.8. Histopathological Examination and IHC Staining Analysis

Liver histology was assessed using hematoxylin and eosin (H&E)-stained paraffin-embedded liver sections (5 μm thick). Images were captured at 20× magnification (0.4942 microns per pixel) using the Snapshot tool in the Aperio ImageScope version 12.4.6. The fat droplet area was quantified using the ImageJ version 1.54h (NIH, Bethesda, MD, USA). The image analysis procedure was as follows. Captured images were first converted to 32-bit grayscale. A consistent threshold was then applied across all samples to generate binary (thresholded) images, where lipid droplets appeared as clear spaces and were highlighted in red. The same thresholding parameters were used uniformly for all images to ensure consistency. The percentage of the fat droplet area was calculated as (area of clear (non-stained) space/total tissue area) × 100.

IHC staining was performed by incubating sections with primary antibodies (1:100 dilution for CCL2 and p65; 1:400 dilution for CD11b) at 4 °C overnight. Bound antibodies were detected using Post Primary (rabbit anti-mouse IgG), Novolink^TM^ Polymer (anti-rabbit poly-horseradish peroxidase IgG), and 3,3′-diaminobenzidine chromogen, following the manufacturer’s protocol (Novolink^TM^ Polymer Detection Systems, Leica Biosystems, Wetzlar, Germany). ImageJ (Media Cybernetics, Bethesda, MD, USA) was used for IHC analysis. The stained area percentage was calculated as (brown-stained area/total tissue area) × 100. The stained cell percentage was calculated as (number of brown-stained cells/total number of cells) × 100. A total of 100 cells were counted per field.

### 2.9. Statistical Analysis

Data were presented as the mean ± standard error. A one-way analysis of variance (ANOVA) followed by Tukey’s post hoc test was conducted using the Prism version 10.2.0. A *p*-value < 0.05 was considered statistically significant.

## 3. Results

### 3.1. 2,5-DHBA Reduced OA-Induced Lipid Accumulation in Hepatocytes

**Figure 1:**
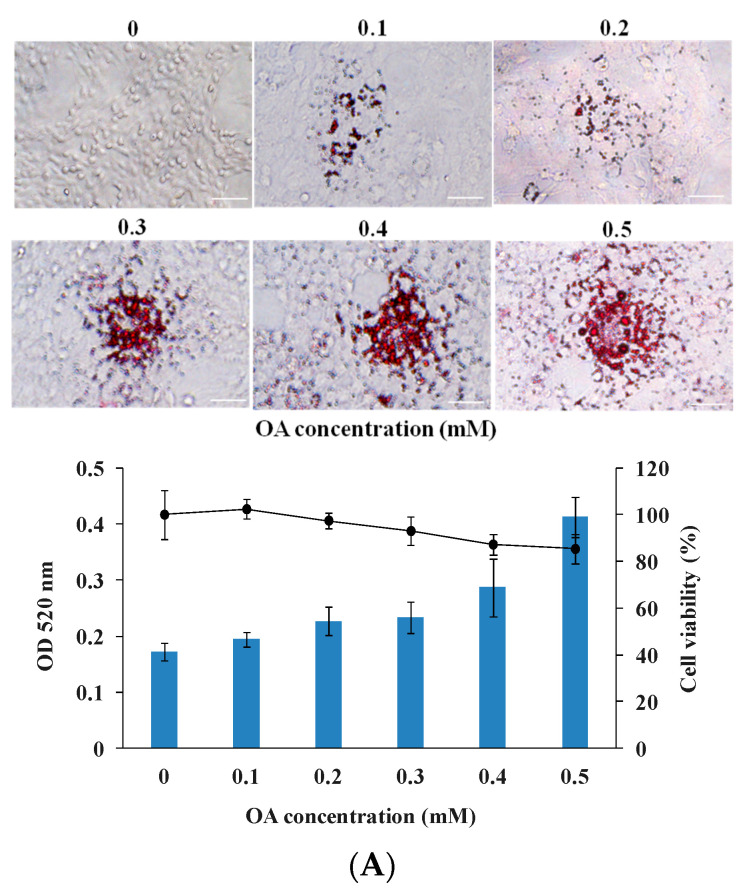

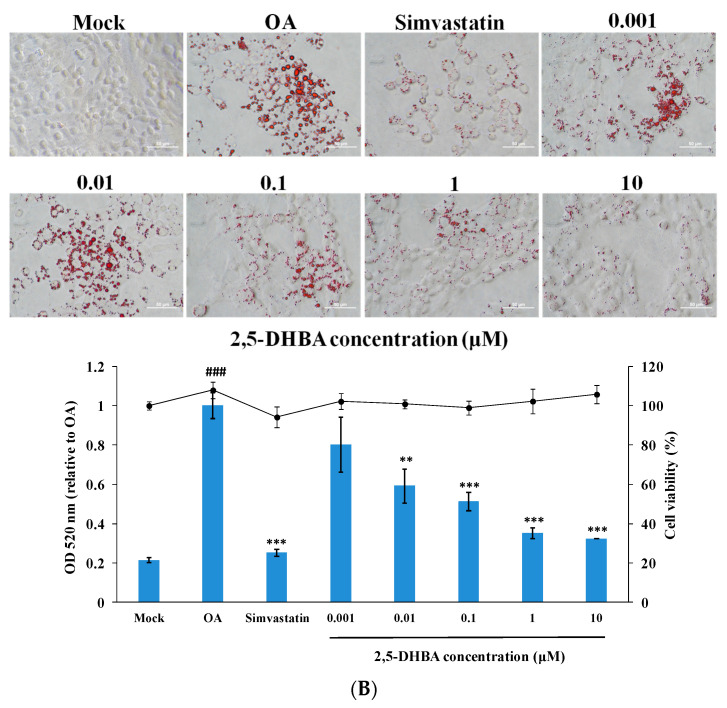
Effects of 2,5-DHBA on OA-induced lipid accumulation in HepG2 cells. (**A**) Dose response of OA. HepG2 cells were treated without or with various concentrations of OA for 24 h. (**B**) Dose response of 2,5-DHBA. HepG2 cells were treated with different concentrations of 2,5-DHBA in the presence of 0.5 mM OA for 24 h. Simvastatin (10 μM) was used as a positive control. Cells were stained with Oil Red O and imaged using a camera and optical microscope at 200× magnification (**top panel**). Scale bar = 50 μm. Representative images are shown. Quantitative analysis of lipid accumulation was performed by measuring Oil Red O content at 520 nm (**bottom panel**). Cell viability was assessed using CCK-8 assay (line graph). Values are mean ± standard error (*n* = 3). ### *p* < 0.001, compared to mock. ** *p* < 0.01 and *** *p* < 0.001, compared to OA.

The effects of 2,5-DHBA on lipid accumulation were initially evaluated using an OA-induced HepG2 cell model, which mimics steatotic hepatocytes in MASLD pathogenesis [24]. HepG2 cells were stimulated with varying concentrations of OA, and lipid accumulation was assessed using Oil Red O staining. As shown in Figure 1A, OA induced dose-dependent lipid accumulation in hepatocytes without affecting cell viability. Based on these results, 0.5 mM OA was selected for subsequent experiments.

Next, HepG2 cells were treated with various concentrations of 2,5-DHBA in the presence of 0.5 mM OA, with simvastatin (10 μM) used as a positive control. As illustrated in Figure 1B, simvastatin significantly reduced OA-induced lipid accumulation without affecting cell viability. Similarly, 2,5-DHBA attenuated OA-induced lipid accumulation in a dose-dependent manner while maintaining cell viability. These findings indicate that 2,5-DHBA effectively inhibited lipid accumulation in OA-induced HepG2 cells.

### 3.2. 2,5-DHBA Ameliorated HFD-Induced Hepatic Lipid Accumulation in Mice

**Figure 2:**
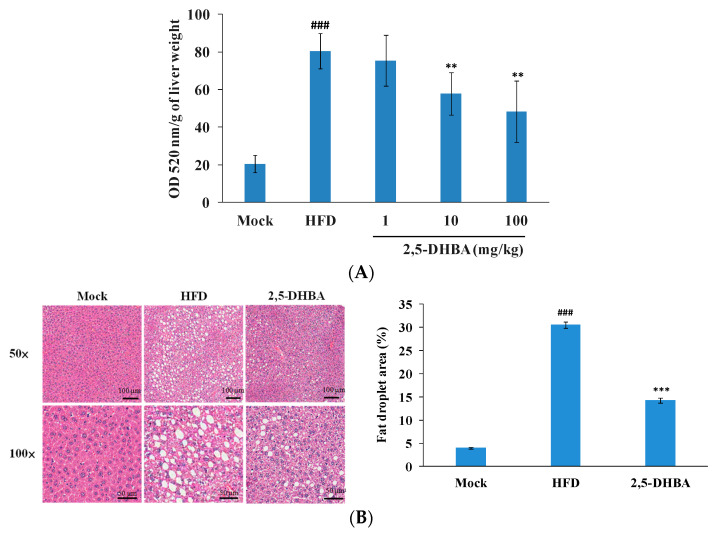
Effects of 2,5-DHBA on HFD-induced hepatic lipid accumulation in mice. (**A**) Short-term experiment. Mice were fed an HFD and orally administered various dosages of 2,5-DHBA for 7 days. Mice in the mock group were fed a normal diet. On day 7, mice were sacrificed, liver tissue was stained with Oil Red O, and lipid content was quantified at 520 nm. Values are mean ± standard error (*n* = 5). ### *p* < 0.001, compared to mock. ** *p* < 0.01, compared to HFD. (**B**) MASLD experiment. Mice were fed an HFD for 4 months. During the fourth month, mice received oral administration of 2,5-DHBA (100 mg/kg) for 4 weeks. Mice in the mock group were fed a normal diet. Liver sections were stained with H&E (**left panel**) and representative images are shown. Quantification of fat droplet area is presented in the right panel. Values are mean ± standard error (*n* = 10). ### *p* < 0.001, compared to mock. *** *p* < 0.001, compared to HFD.

To assess whether 2,5-DHBA could reduce lipid accumulation in vivo, we initially conducted a short-term study to evaluate its dose response in mice. Mice were fed an HFD and orally administered 2,5-DHBA at doses of 1, 10, and 100 mg/kg for 7 days. These doses were selected based on the reported oral lethal dose at 50% (LD_50_) of 2,5-DHBA in mice (~4500 mg/kg) [25], ensuring that all administered doses remained well within a non-toxic range. The highest dose used (100 mg/kg) corresponded to approximately 2% of the LD_50_. This allowed us to assess a broad therapeutic window and determine both the minimal effective and optimal therapeutic doses. The mock group received a normal diet. Lipid accumulation in liver tissue was assessed using Oil Red O staining. As shown in Figure 2A, the HFD group exhibited significantly increased hepatic lipid accumulation compared to the mock group. Treatment with 2,5-DHBA reduced lipid accumulation in a dose-dependent manner. These results suggest that 2,5-DHBA effectively reduced short-term HFD-induced hepatic lipid accumulation.

We then examined the effects of 2,5-DHBA (100 mg/kg) in a long-term MASLD mouse model. Mice were fed an HFD for four months to mimic obesity-associated MASLD [26]. In the fourth month, the mice were orally administered 2,5-DHBA (100 mg/kg) for an additional four weeks. At the end of the experiment, body weight, liver weight, and serological parameters were measured. Table 1 shows that there were no significant differences between the HFD and 2,5-DHBA groups, except in body weight. Liver tissue was then examined histologically. As shown in Figure 2B, the mock group displayed orderly hepatic cords and normal hepatocyte structures. In contrast, the HFD group exhibited clear hepatic steatosis, characterized by disordered cords and ballooned hepatocytes with numerous vacuoles of varying sizes. In the 2,5-DHBA group, steatosis was markedly reduced, with fewer and smaller vacuoles. A previous study has shown that ballooned hepatocytes in MASLD are largely filled with lipid droplets, and their accumulation leads to vacuolation [27]. Therefore, we quantified the vacuole area to assess hepatic fat accumulation. Compared to the mock group, the HFD group had a significantly increased vacuole area (Figure 2B). Treatment with 2,5-DHBA significantly reduced this area. These findings suggest that 2,5-DHBA ameliorates HFD-induced hepatic lipid accumulation in MASLD.

### 3.3. Transcriptomic Analysis of 2,5-DHBA-Regulated Gene Expression in MASLD Livers

**Figure 3:**
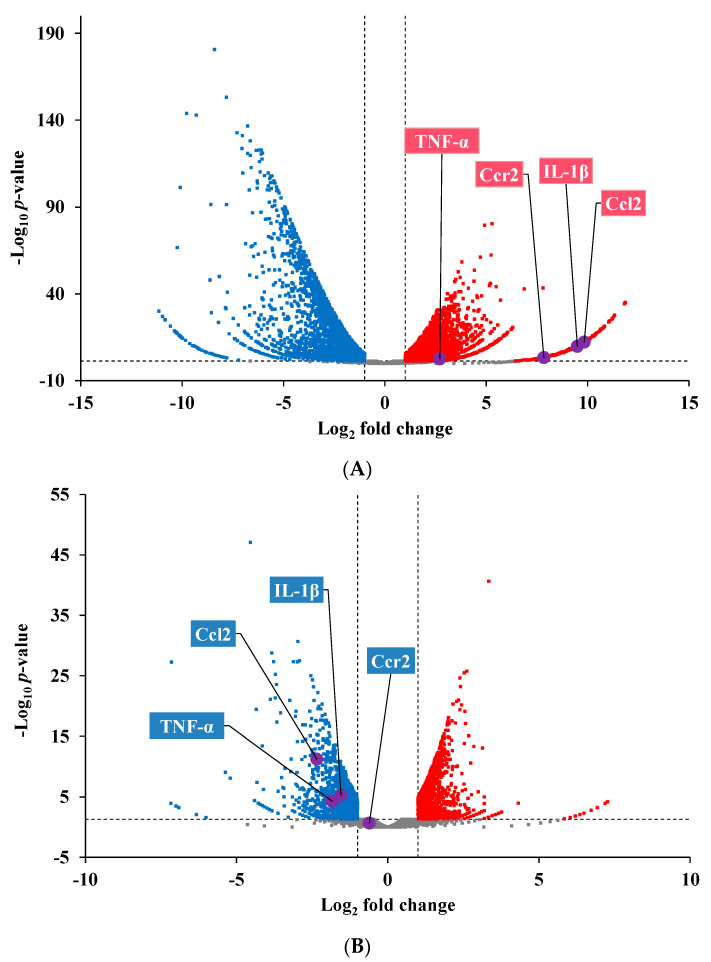
Volcano plot of the expression of genes in the HFD (**A**) and 2,5-DHBA groups (**B**). Red dots indicate genes with a fold change ≥2 and statistically significant differences (adjusted *p* < 0.05). Blue dots represent DEGs with a fold change ≤−2. Gray dots indicate genes without statistically significant changes. The Y-axis shows the − Log_10_ *p*-value, and the X-axis shows the Log_2_ fold change. Dotted horizontal and vertical lines indicate significance thresholds. IL-1β, TNF-α, Ccl2, and Ccr2 are labeled on the plot.

**Figure 4:**
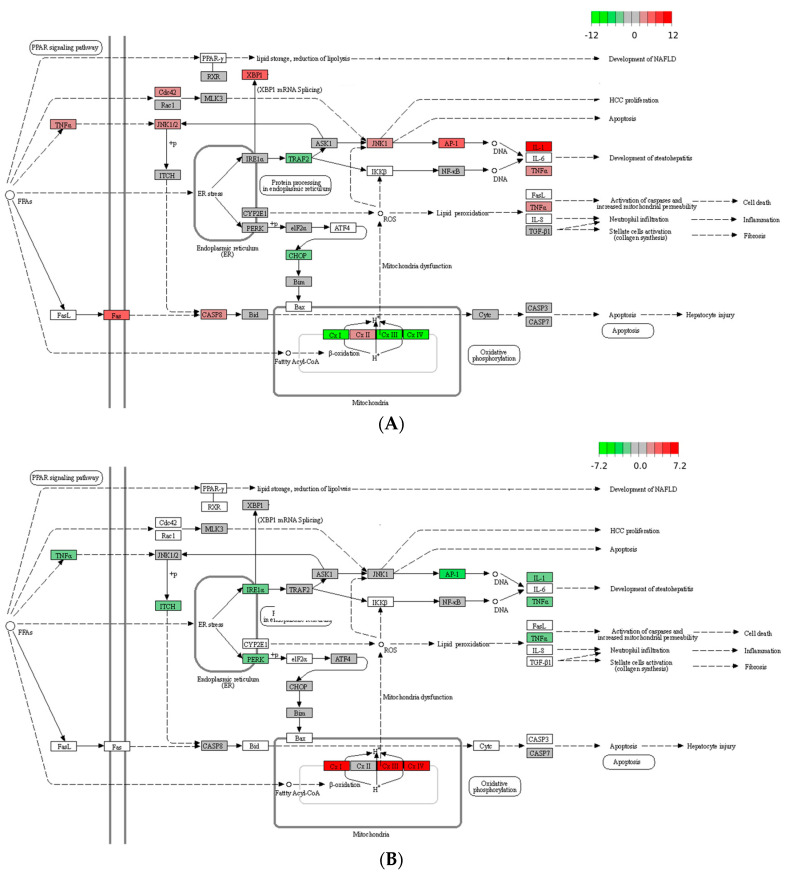
KEGG pathway maps of MASLD affected by HFD and 2,5-DHBA. DEGs affected by HFD (**A**) and 2,5-DHBA (**B**) are shown. Red frames represent upregulated DEGs, and green frames represent downregulated DEGs. Fold changes are indicated by the color scale (top).

To investigate how 2,5-DHBA ameliorated HFD-induced hepatic lipid accumulation, we conducted an RNA-Seq analysis on liver samples. A total of 13,375 transcripts were detected. The volcano plots in Figure 3 illustrate the expression of genes in the HFD and 2,5-DHBA groups. Compared to the mock group, the HFD significantly altered the expression of 8908 DEGs. Treatment with 2,5-DHBA altered 5629 DEGs relative to the HFD group. These DEGs were analyzed using NetworkAnalyst version 3.7 to identify significantly affected biological pathways and potential targets. A total of 172 KEGG pathways were significantly altered by the HFD, and 115 KEGG pathways were affected by 2,5-DHBA. Table 2 lists the top 15 pathways altered by the HFD and 2,5-DHBA. Eleven of these pathways were commonly regulated, primarily involving metabolism, genetic information processing, and MASLD-related disease pathways. Detailed KEGG maps of the MASLD pathways affected by the HFD and 2,5-DHBA are shown in Figure 4. The HFD significantly upregulated proinflammatory genes, such as interleukin-1 (IL-1) and tumor necrosis factor-α (TNF-α), both implicated in steatohepatitis development. In contrast, 2,5-DHBA suppressed their expression. Additionally, while the HFD downregulated genes related to oxidative phosphorylation, 2,5-DHBA upregulated them, suggesting a protective effect on mitochondrial function.

Given the roles of IL-1 and TNF-α in steatohepatitis [28], we further examined chemokine-related gene expression. Table 3 lists 24 chemokine-related genes significantly affected by the HFD or 2,5-DHBA. The HFD upregulated 21 of these genes, while 2,5-DHBA downregulated 18 of these genes. Notably, C-X-C motif ligand 10 (Cxcl10), Cxcl13, and Ccl2 were the most upregulated by the HFD, whereas Cxcl2, Ccl3, and Ccl2 were the most downregulated by 2,5-DHBA. These findings suggest that the CCL2-CCR2 axis plays a key role in MASLD and might be a primary target of 2,5-DHBA.

### 3.4. 2,5-DHBA Competes with CCL2 for Binding to CCR2

**Figure 5:**
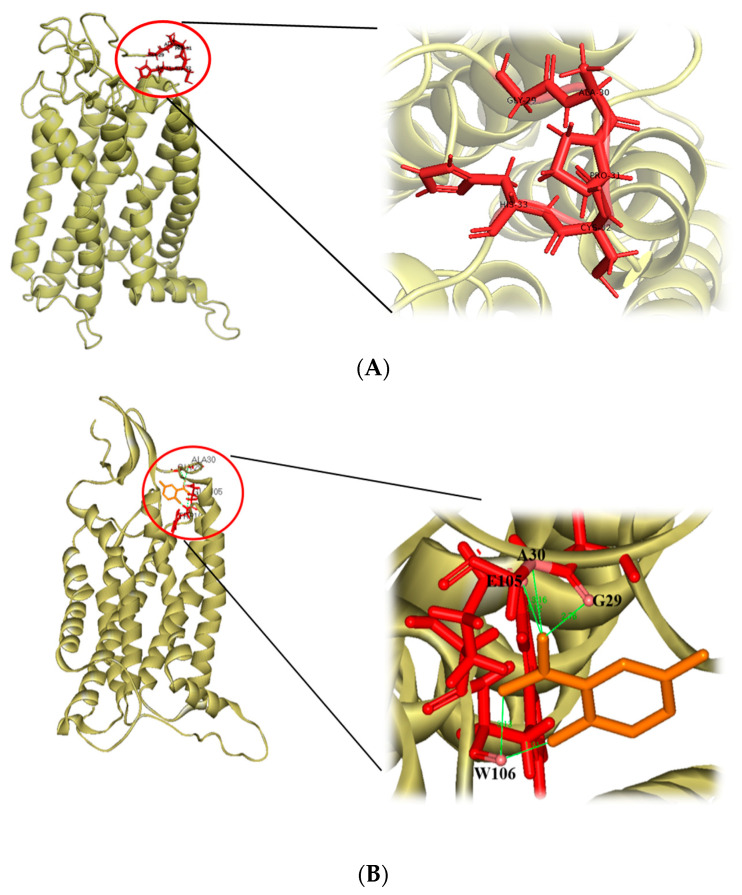
Interaction between 2,5-DHBA and CCR2. (**A**) Structure of CCR2. (**B**) Docking structure of 2,5-DHBA bound to CCR2. The structure of CCR2 is shown as a ribbon. The CCL2-binding region and 2,5-DHBA are shown as red and orange sticks, respectively. Enlarged images highlight key amino acid residues involved in the 2,5-DHBA/CCR2 interaction. Hydrogen bonds between 2,5-DHBA and CCR2 are represented by green lines.

To investigate how 2,5-DHBA affected the CCL2-CCR2 pathway, we performed a docking analysis. The cryo-EM structure of CCL2 bound to CCR2 reveals key hydrophobic and hydrogen bond interactions involving CCR2 residues Gly-29 and His-33 [29]. This binding site was used for the docking of 2,5-DHBA (Figure 5A). As shown in Figure 5B, 2,5-DHBA docked into the CCL2-binding region, forming hydrogen bonds with Gly-29 and Ala-30 at distances of 2.76 and 3.16 Å, respectively. Additionally, hydrogen bonds were formed with Glu-105 (3.12 Å) and Trp-106 (1.91 and 3.13 Å), near the CCL2-binding region. These results suggest that 2,5-DHBA competed with CCL2 for CCR2 binding, potentially inhibiting downstream CCL2-CCR2 signaling. This interaction may underlie the anti-inflammatory effects observed with 2,5-DHBA treatment.

### 3.5. 2,5-DHBA Reduces CCL2 Expression, Nuclear Factor-κB (NF-κB) Activation, and Inflammatory Cell Infiltration in MASLD Mouse Livers

To verify the effects of 2,5-DHBA on CCL2-CCR2 signaling in vivo, IHC staining was performed for CCL2 in liver tissue. Previous studies have shown that increased hepatic CCL2 recruits CCR2-positive monocytes, exacerbating inflammation, fibrosis, and steatosis in MASLD patients [30,31,32]. Therefore, antibodies against CCL2, p65, and CD11b were used in IHC staining to detect the expression of CCL2, the activation of NF-κB, and the infiltration of inflammatory cells, respectively.

**Figure 6:**
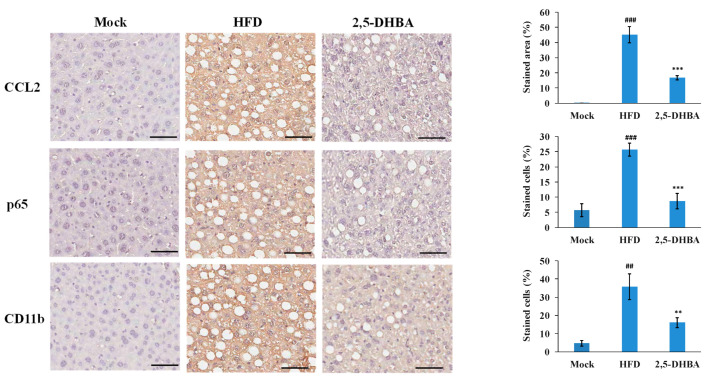
IHC staining analysis of CCL2 expression, p65 activation, and inflammatory cell infiltration in response to 2,5-DHBA treatment. Mice were fed an HFD for 4 months. During the fourth month, mice received 2,5-DHBA (100 mg/kg) orally for 4 weeks. Mice in the mock group were fed a normal diet. Liver sections were stained with antibodies against CCL2, p65, and CD11b. Original magnification: 100×. Scale bar = 50 μm. Representative images are shown. Quantification of the stained areas or cells percentages is shown in the right panel. Values are mean ± standard error (*n* = 10). ## *p* < 0.01, ### *p* < 0.001, compared to mock. ** *p* < 0.01, *** *p* < 0.001, compared to HFD.

As shown in Figure 6, the hepatic CCL2 expression was significantly increased in the HFD group compared to the mock group, confirming the positive correlation between CCL2 and the MASLD animal model. The levels of p65- and CD11b-positive cells were also significantly elevated, indicating the activation of the CCL2-CCR2 pathway. However, 2,5-DHBA treatment significantly reduced the levels of CCL2, as well as the numbers of p65- or CD11b-stained cells. These data suggest that the activation of the CCL2/CCR2 pathway in the MASLD model promoted immune cell recruitment to the liver, contributing to inflammation and disease progression. However, 2,5-DHBA disrupted the CCL2-CCR2 interaction by competing for CCR2 binding, thereby reducing inflammatory cell infiltration and ameliorating MASLD progression.

## 4. Discussion

Nutritional and dietary approaches play a critical role in addressing the growing burden of MASLD [4,5]. The DIRECT PLUS randomized clinical trial reveals that the green Mediterranean diet leads to a twofold increase in intrahepatic fat loss, with participants following this diet exhibiting significantly higher levels of total polyphenols, particularly 2,5-DHBA, in their plasma [16]. These data suggest that a reduction in liver fat may be linked to elevated levels of 2,5-DHBA. However, to date, no studies have directly examined the relationship between 2,5-DHBA and MASLD. In this study, we found that 2,5-DHBA reduced OA-induced lipid accumulation in hepatocytes and ameliorated hepatic lipid accumulation in HFD-fed mice. This is the first study to report the beneficial effects of 2,5-DHBA in improving MASLD.

The pathogenesis of MASLD is a complex process involving impaired lipid metabolism, inflammation, and the generation of reactive oxygen species, all of which contribute to disease progression [33]. Consequently, both metabolic targets, such as peroxisome proliferator-activated receptors (PPARs) and the farnesoid X receptor, and immune targets, such as chemokines and their receptors, have been explored as therapeutic avenues for MASLD [34,35,36,37]. To identify the molecular targets affected by 2,5-DHBA, we performed an RNA-Seq analysis on liver tissue from an MASLD mouse model. Clinical and experimental evidence indicates that the hepatic levels of chemokines and their receptors are elevated in MASLD. For example, the hepatic CCL2 levels are positively correlated with fat accumulation in both MASLD patients and diet-induced mouse models [38]. Notably, the genetic deletion or inhibition of CCL2 or its receptor CCR2 ameliorates hepatic inflammation and fibrosis in these models [38,39]. Blocking the CCL2-CCR2 axis using a CCL2-neutralizing antibody also suppresses macrophage infiltration and reduces inflammation in chronic liver diseases [40]. These findings underscore the critical role of the CCL2-CCR2 axis in MASLD progression [30,32]. In our study, we found that the expression of most chemokine ligand and receptor genes was significantly upregulated by the HFD, consistent with previous studies. Interestingly, 2,5-DHBA downregulated these genes’ expression, particularly CCL2, suggesting that the CCL2-CCR2 axis may be a key target for 2,5-DHBA in mitigating MASLD.

How does 2,5-DHBA affect the CCL2-CCR2 interaction? Cryo-EM images of the CCL2-bound CCR2 structure show that CCL2 binds securely to the extracellular part of CCR2’s transmembrane domain, where residues Gly-29 to His-33 form several hydrophobic and hydrogen bonds with CCL2 [29]. Our docking analysis revealed that 2,5-DHBA formed hydrogen bonds with Gly-29, Ala-30, Glu-105, and Trp-106 of CCR2. We speculated that the hydroxyl group of 2,5-DHBA formed two hydrogen bonds with Trp-106, guiding the molecule into the hydrophobic pocket of CCR2. Its carboxyl group formed a hydrogen bond with Glu-105 to stabilize the position, as well as additional bonds with Gly-29 and Ala-30, thereby competing with CCL2 for CCR2 binding. This competitive inhibition may disrupt downstream CCL2-CCR2 signaling. However, further studies using structure biology tools, such as hydrogen–deuterium exchange mass spectrometry and nuclear magnetic resonance, are warranted to confirm the detailed interactions between 2,5-DHBA and the CCL2/CCR2 complex.

Our data suggest that 2,5-DHBA disrupted the CCL2-CCR2 signaling axis through two complementary mechanisms. First, 2,5-DHBA appeared to compete with CCL2 for binding to CCR2, thereby blocking receptor activation and downstream signaling. Second, 2,5-DHBA suppressed hepatic CCL2 production. Elevated free fatty acids and the activation of toll-like receptors are known to stimulate Kupffer cells to produce CCL2 via NF-κB signaling. CCL2, in turn, plays a central role in recruiting monocytes and macrophages to the liver, amplifying inflammation [38]. Upon binding CCR2, CCL2 also activates both resident and infiltrating macrophages, promoting the release of proinflammatory cytokines and exacerbating hepatic inflammation [41]. This CCL2-CCR2 interaction further stimulates NF-κB signaling, creating a feedforward loop that promotes macrophage infiltration and liver fibrosis [30,32]. In this study, we showed that 2,5-DHBA downregulated hepatic CCL2 expression, inhibited NF-κB activation, and reduced macrophage infiltration. These findings support our hypothesis that 2,5-DHBA interferes with both ligand–receptor interaction and proinflammatory signaling pathways, thereby mitigating MASLD progression.

Nutritional and dietary strategies remain essential in managing the rising prevalence of MASLD [4,5]. However, clinical trials investigating low-fat or low-sugar diets in MASLD patients have shown heterogeneous outcomes. Similarly, dietary supplements, such as antioxidants, probiotics, and polyunsaturated fatty acids, have produced inconsistent results [42]. Some foods or components have shown efficacy in diet-induced MASLD animal models or cell models. For instance, *Prunus domestica* L. subsp. *syriaca* extract reduces lipid accumulation in OA-induced HepG2 cells by downregulating lipogenic and oxidative stress-related genes and reducing the reactivated oxygen species production [43]. Isoschaftoside from fig leaves suppresses M1 macrophage marker expression, suggesting its role in modulating macrophage polarization and reducing lobular inflammation in HFD-induced MASLD mice [44]. Myricitrin, a flavonoid in fruits and vegetables, lowers the atherogenic index and cardiovascular risk factors and inhibits hepatic 3-hydroxy-3-methylglutaryl-CoA reductase and acyl-CoA:cholesterol acyltransferase activity, improving hypercholesterolemia and MASLD in mice [45]. Epigallocatechin gallate protects against MASLD by modulating gut microbiota dysbiosis, intestinal barrier dysfunction, and inflammation [46]. Millet bran protein hydrolysate inhibits fatty acid uptake through PPARγ activation, alleviating hepatic steatosis and reducing lipid accumulation [47]. *Auricularia polytricha* aqueous extract reduces inflammation, oxidative stress, and lipid deposition in MASLD models [48]. Nevertheless, the clinical efficacy of these foods or constituents in MASLD patients remains to be investigated. In this study, we conducted a bedside-to-bench translational investigation of 2,5-DHBA, a polyphenol metabolite that has been positively associated with reduced intrahepatic fat in the DIRECT PLUS trial. Our findings provide evidence that 2,5-DHBA ameliorates lipid accumulation in hepatocytes and in HFD-induced MASLD mice, likely through the modulation of the CCL2-CCR2 axis.

## 5. Conclusions

The green Mediterranean diet represents a promising nutritional strategy for MASLD management. While food chemistry research has traditionally focused on the compounds present in foods, less attention has been paid to their bioactive metabolites. In this study, we identified 2,5-DHBA, a polyphenol metabolite that is elevated in individuals following a green Mediterranean diet, as a key modulator. We demonstrated that 2,5-DHBA competed with CCL2 for binding to CCR2, thereby altering CCL2 production, suppressing NF-κB activation, and reducing inflammatory cell infiltration, ultimately slowing MASLD progression in HFD-fed mice. This is the first study to report the health benefits of 2,5-DHBA in MASLD. Our findings suggest that 2,5-DHBA has potential as a nutraceutical supplement for the prevention or treatment of MASLD in the future.

## Figures and Tables

**Table 1:** Body weight gain, food intake, calorie intake, liver weight, and serological parameters in HFD-fed mice treated with 2,5-DHBA.

	HFD	2,5-DHBA	*p*-Value
Body weight gain (g)	23.00 ± 2.69	27.86 ± 2.57 *	0.022
Food intake (g/day)	2.60 ± 0.18	2.20 ± 0.34	0.086
Calorie intake (kcal)	13.26 ± 0.92	11.22 ± 1.76	0.086
Liver weight (g)	1.20 ± 0.25	1.36 ± 0.11	0.236
Liver weight/body weight (%)	5.20 ± 0.50	4.90 ± 0.20	0.266
GOT (U/L)	274.40 ± 98.38	243.60 ± 90.87	0.621
Blood urea nitrogen (mg/dL)	22.70 ± 3.06	20.34 ± 3.23	0.270
Creatinine (mg/dL)	0.46 ± 0.10	0.51 ± 0.04	0.294
Cholesterol (mg/dL)	117.60 ± 15.49	123.60 ± 13.46	0.532
Triglyceride (mg/dL)	125.80 ± 35.25	138.80 ± 28.31	0.538

* *p* < 0.05, compared to HFD group.

**Table 2:** Biological pathways significantly altered by HFD and 2,5-DHBA in the liver tissue of MASLD mice.

KEGG Pathway	Observed ^1^/Total ^2^	*p*-Value
**HFD-altered**		
Chemical carcinogenesis—reactive oxygen species	167/175	1.64 × 10^−26^
Oxidative phosphorylation	105/224	5.82 × 10^−19^
Thermogenesis	157/188	1.05 × 10^−17^
Non-alcoholic fatty liver disease	114/127	7.90 × 10^−17^
Protein processing in endoplasmic reticulum	120/144	3.93 × 10^−15^
Peroxisome	69/74	3.98 × 10^−14^
Biosynthesis of cofactors	107/375	4.78 × 10^−14^
Ubiquitin-mediated proteolysis	97/133	2.59 × 10^−10^
Carbon metabolism	83/366	2.77 × 10^−10^
Fluid shear stress and atherosclerosis	94/99	5.75 × 10^−9^
Proteasome	38/49	1.59 × 10^−8^
Spliceosome	86/123	1.98 × 10^−8^
Ribosome	108/143	4.64 × 10^−8^
Apoptosis	86/110	5.46 × 10^−8^
Autophagy—animal	89/132	5.91 × 10^−8^
**2,5-DHBA-altered**		
Ribosome	109/143	4.07 × 10^−24^
Chemical carcinogenesis—reactive oxygen species	123/175	5.59 × 10^−22^
Oxidative phosphorylation	84/224	1.04 × 10^−19^
Thermogenesis	121/188	6.03 × 10^−19^
Non-alcoholic fatty liver disease	86/127	1.51 × 10^−15^
Proteasome	30/49	2.93 × 10^−8^
mTOR signaling pathway	70/117	1.11 × 10^−7^
Chemical carcinogenesis—DNA adducts	43/129	3.21 × 10^−7^
Autophagy—animal	63/132	5.89 × 10^−7^
Biosynthesis of cofactors	65/375	1.91 × 10^−6^
Drug metabolism—other enzymes	44/132	2.61 × 10^−6^
Apoptosis	59/110	3.24 × 10^−6^
Protein processing in endoplasmic reticulum	69/144	1.36 × 10^−5^
TNF signaling pathway	49/94	2.19 × 10^−5^
Fluid shear stress and atherosclerosis	60/99	2.66 × 10^−5^

^1^ Number of DEGs altered by HFD or 2,5-DHBA. ^2^ Number of genes involved in the pathway.

**Table 3:** List of chemokine-related genes significantly affected by HFD and 2,5-DHBA in the liver tissue of MASLD mice.

Symbol	Description	Fold Change			*p*-Value	
		HFD	2,5-DHBA		HFD	2,5-DHBA
Ccl2	chemokine (C-C motif) ligand 2	912.556	−5.086		9 × 10^−14^	8 × 10^−14^
Ccl3	chemokine (C-C motif) ligand 3	158.494	−5.344		0.0021	6 × 10^−5^
Ccl4	chemokine (C-C motif) ligand 4	67.816	−1.529		0.0158	0.4305
Ccl7	chemokine (C-C motif) ligand 7	96.451	−3.246		0.0246	0.0143
Ccl24	chemokine (C-C motif) ligand 24	−2.159	1.345		0.0031	0.1373
Ccl25	chemokine (C-C motif) ligand 25	−3.644	−1.031		0.0018	1
Ccr1	chemokine (C-C motif) receptor 1	96.451	−2.791		0.0246	0.0277
Ccr2	chemokine (C-C motif) receptor 2	230.082	−1.528		2 × 10^−4^	0.1265
Ccrl2	chemokine (C-C motif) receptor-like 2	5.131	−1.901		0.0084	0.0189
Ccr5	chemokine (C-C motif) receptor 5	1.596	−1.833		0.2124	0.0085
Cx3cl1	chemokine (C-X3-C motif) ligand 1	48.725	1.812		0.0226	0.0214
Cx3cr1	chemokine (C-X3-C motif) receptor 1	−2.328	2.013		0.0195	0.0105
Cxcl1	chemokine (C-X-C motif) ligand 1	37.857	−4.968		6 × 10^−65^	8 × 10^−26^
Cxcl2	chemokine (C-X-C motif) ligand 2	273.035	−6.946		3 × 10^−5^	3 × 10^−8^
Cxcl9	chemokine (C-X-C motif) ligand 9	9.087	−1.484		6 × 10^−11^	0.02157
Cxcl10	chemokine (C-X-C motif) ligand 10	2344.319	−2.635		2 × 10^−27^	6 × 10^−9^
Cxcl11	chemokine (C-X-C motif) ligand 11	129.859	1.273		0.0096	0.5018
Cxcl12	chemokine (C-X-C motif) ligand 12	2.676	−1.387		2 × 10^−10^	0.0232
Cxcl13	chemokine (C-X-C motif) ligand 13	1275.269	−1.769		1 × 10^−17^	0.0012
Cxcl14	chemokine (C-X-C motif) ligand 14	139.404	−2.587		0.0057	0.0127
Cxcl16	chemokine (C-X-C motif) ligand 16	2.159	1.094		0.0033	0.6099
Cxcr2	chemokine (C-X-C motif) receptor 2	244.34	−3.132		1 × 10^−4^	2 × 10^−4^
Cxcr4	chemokine (C-X-C motif) receptor 4	63.043	−1.166		0.0132	0.8477
Cxcr6	chemokine (C-X-C motif) receptor 6	63.043	−2.117		0.0132	0.0187

## Data Availability

Data available on request from the authors.

## Abbreviations

The following abbreviations are used in this manuscript:

CCK-8	Cell Counting Kit-8
CCL2	Chemokine (C-C motif) ligand 2
CCR2	Chemokine (C-C motif) receptor 2
Cxcl10	C-X-C motif ligand 10
DEGs	Differentially expressed genes
2,5-DHBA	2,5-Dihydroxybenzoic acid
DIRECT PLUS	Dietary Intervention Randomized Controlled Trial Polyphenols Unprocessed
DMEM	Dulbecco’s modified Eagle’s medium
DMSO	Dimethyl sulfoxide
H&E	Hematoxylin and eosin
HFD	High-fat diet
IHC	Immunohistochemical
IL-1	Interleukin-1
KEGG	Kyoto Encyclopedia of Genes and Genomes
LD_50_	Lethal dose at 50%
MASLD	Metabolic dysfunction-associated steatotic liver disease
NF-κB	Nuclear Factor-κB
OD	Optical density
PBS	Phosphate-buffered saline
PPARs	Peroxisome proliferator-activated receptors
RNA-Seq	RNA sequencing
TNF-α	Tumor necrosis factor-α
